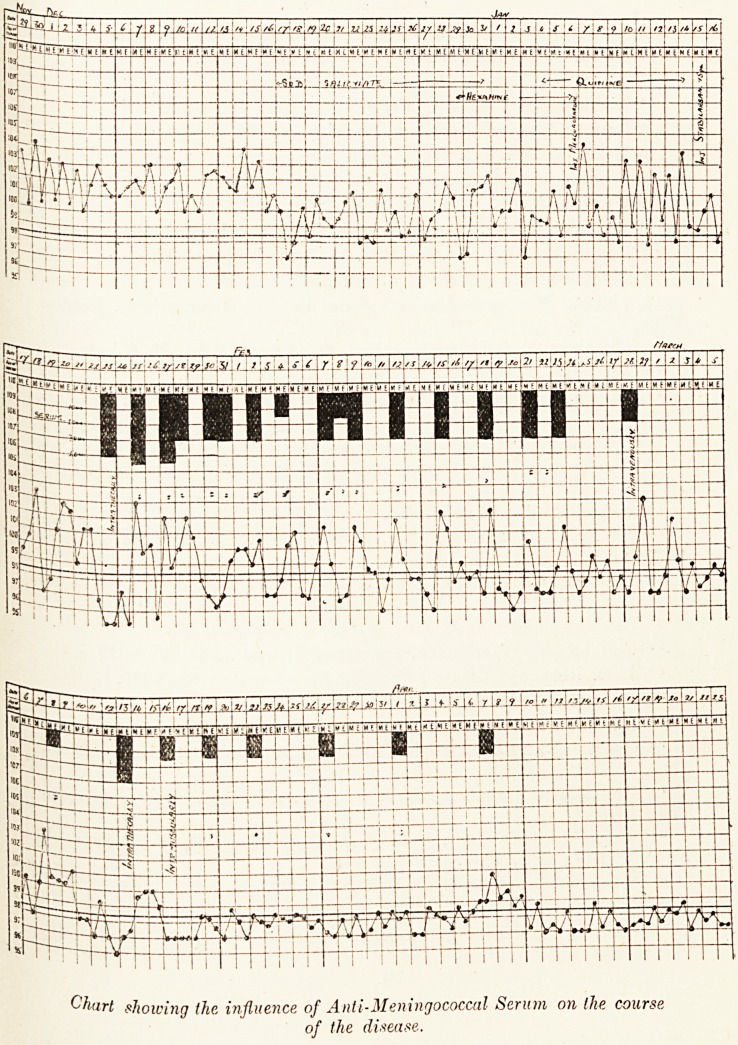# Meningococcal Septicæmia with a Report of a Case

**Published:** 1928

**Authors:** C. Bruce Perry

**Affiliations:** Colston Research Fellow in the University Centre of Cardiac Research, and Medical Registrar, Bristol General Hospital


### MENINGOCOCCAL SEPTICÆMIA.

### WITH REPORT OF A CASE.

BY

C. Bruce Perry, M.B., M.R.C.P.,

*Colston Research Fellow in the University Centre of Cardiac Research, and Medical Registrar, Bristol General Hospital.*

---

Although the case to be described presents no new points, the rarity of the disease in this country, which is more frequently recognised in America and on the Continent, makes it worthy of record.

### Case Report.

*History of Present Illness.*—On December 17th, A. B., male, aged 12, was admitted to the Bristol General Hospital, with the history that five weeks previously he was suddenly taken ill with a " chill." This was accompanied by a succession of crops of small red papules distributed on the face, limbs, mostly on the extensor surfaces, and on the abdomen. These faded in about four days, some leaving a faint stain. Three weeks ago he began to have pains in his knees and arms. For the last three weeks his temperature had been irregular. (It was not taken regularly before this time.)

*Previous History.*—" Consumptive bowels " as a baby. Mumps and measles several years ago.

*Family History.*—Father and mother alive and healthy. Five other children healthy. No miscarriages.

*Condition on Examination.*—Patient rather pale. Appetite good, sleeps well, bowels regular, micturition normal. Teeth good ; tongue furred.

Generalised red maculo-papular eruption. In one or two places drying pustules, possibly due to scratching.

Heart not enlarged. Faint systolic bruit all over præcordium. No abnormal physical signs in lungs.

Abdomen normal in appearance. Spleen just palpable, below left costal margin. Liver not palpable.

No head retraction or Kernig's sign. Knee-jerks present and plantar reflex flexor. Pupils react to light and accommodation. Optic discs and fundi normal.

Urine: specific gravity 1016, reaction acid. No albumin, blood or sugar.

*Subsequent Progress.*—December 19th, 1927. Rash most marked in febrile periods; thought by dermatologist to resemble a drug rash. Patient quite comfortable in afebrile periods. Treated with salicylates.

December 29th, 1927.—Condition unchanged. Hexamine being given by mouth. Blood culture negative.

January 2nd, 1928.—Leucocytes 8,000 per c.mm.; polymorphonuclears 66 per cent. No malarial parasites seen in films.

January 4th, 1928.—Quinine given by mouth.

January 5th, 1928.—Blood culture negative. Blood shows no agglutination with Bacillus typhosus, paratyphosus A and B or with micrococcus melitensis.

January 6th, 1928.—Given 5 c.c. of 0·5 per cent. solution of mercurochrome intravenously.

January 9th, 1928.—Urine: no abnormality in deposit. Culture sterile.

January 16th, 1928.—Condition unchanged, given 0·15 gm. stabilarsan intravenously.

January 21st, 1928.—Patient vomited several times and complained of headache.

January 23rd, 1928.—Patient much worse. Lies on side with knees drawn up. Head retraction and Kernig's sign present. Lumbar puncture. Fluid under increased pressure and turbid. Laboratory report: "Protein much increased. Chlorides 0·585 per cent. Many polymorphonuclear leucocytes. Intracellular Gram-negative diplococci. Culture shows meningococci." Intrathecal anti-meningococcal serotherapy instituted.

January 27th, 1928.—Patient very ill. Has severe headache. Delusions at times.

February 22nd, 1928.—General condition improved. Cerebro-spinal fluid report: Protein 0·1 per cent. Globulin increased. Chlorides 0·695 per cent. Cells 162 per c.mm., polymorphonuclears and lymphocytes. A few cocci still present. White cell count: 7,000 per c.mm.; polymorphonuclears, 58 per cent.

February 27th, 1928.—Optic discs show slight optic neuritis both sides. General condition better. Meningeal symptoms nearly clear, but fever and rash continue.

March 13th, 1928.—Cerebro-spinal fluid clear. Protein 0·15 per cent. Chlorides 0·695 per cent. Cells mainly mononuclears. No organisms seen. Culture sterile. Serum given intravenously, followed by severe reaction.

March 16th, 1928.—Serum given intravenously.

March 22nd, 1928.—No meningeal symptoms remain. Temperature normal. No rash.

March 31st, 1928.—Some pain on micturition. Urine: reaction acid. Deposit: large numbers of red blood cells, fair number of white cells. No casts.

April 12th, 1928.—Patient apparently quite well. Urine: deposit shows small number of pus and red blood cells. No casts. No tubercle bacilli. Culture sterile.

April 20th, 1928.—No abnormality in urine.

April 23rd, 1928.—Patient discharged well. When last seen, ten days after discharge, he remained well, and free from any evidence of organic disease. The cause of the hæmaturia was not solved; possibly it was due to the administration of serum.

### SUMMARY OF LITERATURE.

The first record of the isolation of the meningococcus from the blood was made by Osler[1] in 1898 from a case of meningococcal meningitis. In 1902 Salomon[2] reported a case of primary meningococcal septicæmia with intermittent fever and a maculopapular eruption, which terminated in meningitis after two months and ultimately recovered.

In 1906 Andrewes[3] reported a case of meningococcal septicæmia, which proved fatal in four days, with no sign of meningeal involvement. In 1908 Liebermeister[4] described a case of septicæmia, due to the meningococcus, that recovered without meningitis. Since that date many cases of meningococcal septicæmia and of extrameningeal meningococcal infections have been reported. Cecil and Soper[5] record a case of meningococcal endocarditis, as do also Warfield and Walker[28] and Schottmüller.[29] Herrick[6] reports cases of meningococcal empyema and of bronchopneumonia, both without meningitis. Findlay[7] records a case of polyarthritis in which meningococci were found post-mortem in the joints. Jacobitz[30] describes cases of pneumonia due to the meningococcus.

It is now generally considered that all cases of meningococcal meningitis begin with a short phase of bacteriæmia. Herrick,[8] in 315 cases of meningitis, was able to make the diagnosis before the onset of meningitis by means of blood cultures in 40 per cent. He found that the average length of this invasive stage was forty-eight hours. Blackfan[9] states that once the infection is localised in the meninges there is usually no bacteriæmia.

Herrick, writing in Osler and McCrae's *Modern Medicine*,[21] divides a meningococcal infection into three stages :—

1. A stage of coryza, tonsillitis and pharyngitis. This may not progress and the patient may become a chronic carrier.

2. An invasive stage of bacteriæmia, usually lasting forty-eight hours, which may be prolonged for days or weeks.

3. A metastatic stage. In 90 per cent. the dominant metastasis is in the meninges, but it may occur in the joints, in the pericardium, endocardium, lungs or epididymis.

According to Goodall and Washbourn[22] septicæmic forms of meningococcal infection may be of three types :—

(*a*) Fulminating types, rapidly fatal with no localisation.

(*b*) Abortive types with rash and fever, lasting two or three days to a week and terminating in recovery, with no meningitis.

(*c*) Chronic types which persist for weeks or months and may or may not be complicated by meningitis. Rolleston[23] agrees with this.

Boulanger-Pilet[10] describes seven types of pure septicæmia :—

1. A pseudo-malarial type with intermittent fever, an eruption usually maculopapular, but sometimes morbilliform, scarlatiniform or herpetiform, waxing and waning with the fever; transitory joint and muscle pains, and a polymorphonuclear leucocytosis.

2. A purpuric type which may be rapidly fatal.

3. A typhoid type.

4. An articular type with arthritis which may be purulent.

5. Mixtures of the above type, *e.g.* beginning as a typhoid type and becoming pseudo-malarial.

6. A type with abnormal termination. The infection may become localised in unusual situations, causing such lesions as pleurisy, endocarditis and iridocyclitis.

7. A "forme fruste" corresponding to Goodall and Washbourn's abortive type.

For these infections the French have coined the word "Meningococcémies."

Apart from the fulminating cases proving rapidly fatal in a few hours, often with a purpuric eruption (Herrick),[6] the usual course is that of the "type pseudo-palustre" of Boulanger-Pilet.[10] The main clinical features are an irregular, intermittent fever associated with a maculo-papular eruption and fleeting joint and muscle pains. In the apyrexial intervals the patient appears perfectly well (Herrick,[6] Neergard,[14] Bloedorn,[11] Vignot[12]). The rash is in some cases described as being like erythema nodosum (Liegois and Foulon[13]). There may be no rash (Vaucher and Schmid[15]) or no joint or muscle pains (Gandy and

Boulanger-Pilet[17], Gandy and Deguinard[19]); or merely irregular fever with headache and malaise (Netter,[16] Debré, Ravine and de Pfeffel[18]). The spleen is often palpable and there is a polymorphonuclear leucocytosis somewhere in the neighbourhood of 15,000 per c.mm. The case described is unusual in that at the times the blood was examined the highest number of leucocytes found was 8,000 per c.mm. A blood culture, unless made on special media, may be negative, as in the case reported (see also Bloedorn,[11] Neergard,[14] Gandy and Deguinard[19]). There may be slight transient albuminuria (Gandy and Boulanger-Pilet[17]); or, more rarely, a subacute nephritis leaving a persistent albuminuria (Gandy and Deguinard[19]). There may be a transient epididymo-orchitis.[19]

These cases usually terminate in a meningitis, and, as in the case described by Netter,[16] may last as long as 102 days. Less often they may terminate without localisation, a general septicæmia persisting throughout the whole course of the disease; or there may be localisation in some abnormal site causing iridocyclitis, pneumonia, endocarditis,[10] etc. More rarely this condition may supervene upon an apparently cured meningitis.[20] There may be relapses and recurrences of the meningitis.[19] In the pure septicæmias and before the onset of meningitis the cerebro-spinal fluid shows no changes ([17, 19, 15]).

The disease appears to occur more commonly in children. The diagnosis may be made, in the opinion of some authors,[19] from the intermittent fever, rash and joint pains, even in the presence of negative blood cultures. In the case of negative blood cultures confirmation of the diagnosis may be obtained by means of agglutination or complement deviation reactions ([16]). The differential diagnosis has to be made from malaria, abortus fever, typhoid, and at first the condition may, as in this case, be diagnosed as acute rheumatism. The rash may easily be mistaken for a drug rash. Often the diagnosis remains uncertain, as in the case described, until the occurrence of meningitis.[14]

The pathogenesis is obscure, but such cases as those reported by Gandy and Deguinard,[19] in which a recrudescence of meningitis occurred, and that of Lauvergne and Carrot,[20] in which septicæmia followed an apparently cured meningitis and the exhibition of an autogenous vaccine, favour the view that the process is not a septicæmia in the generally accepted sense, but that continually repeated invasions of the circulation occur from some isolated focus in or near the meninges. There is, however, no proof as yet forthcoming of this view, which is therefore entirely speculative.

As regards treatment, the efficiency of different methods is debated. Many cases, as the present one, appear to have been cured by the exhibition of anti-meningococcal serum, either subcutaneously, intra-muscularly or intravenously ([19, 11, 6, 18, 13, 31]). Others have found serotherapy absolutely ineffectual, and, of these, some record success with autogenous or stock vaccines in resistant cases. Courtois-Suffit and Garnier's case[26] terminated fatally after the administration of serum and vaccines had proved useless. Vaucher and Schmid[15] consider that their patient was cured by the injection of 10 c.c. of sterile milk, whereas Netter[16] found this inefficacious, as also uroformin, colloidal metals, quinine and novarsenobenzol. His patient was cured by the injection of sterile pus from a fixation abscess. Good effects following the production of a fixation abscess are recorded by other workers.[20]

Luton,[24] discussing the diminished value of serotherapy in meningococcal infections since the war, attributes it to the increase in prevalence of meningococcus B (or type II. of Gordon), which is known to be intractable to serotherapy. He describes recovery from meningitis and from septicæmia following the use of a meningococcal endoprotein. At first he was inclined to regard the action of this as specific. He now considers it to be due to non-specific protein shock. Hermann and Lifschitz[25] record a case recovering spontaneously after the ineffective exhibition of quinine, iron and arsenic. This observation may be ranked with that of Monziols and Loiseleur,[27] who record the complete cure of a patient following the withdrawal of blood for a blood culture with no other treatment.

### SUMMARY OF CASE.

The striking features of the case described were :—

1. The difficulty of diagnosis until the development of the meningitis. Acute rheumatism, malaria, "abortus" fever and miliary tubercle were among the diagnoses considered at different times.

2. At no time when the white cells were counted was there a marked leucocytosis.

3. The prolonged course of the pyrexia (see chart). This persisted for ten weeks before, and for another seven weeks after meningitis developed.

4. Intrathecal injection of anti-meningococcal serum was without effect on the pyrexia, although improving the meningitis, which ran a very protracted course. Both were promptly arrested after the intravenous and intramuscular injection of polyvalent anti-meningococcal serum.

I should like to express my gratitude to Dr. Rogers for the cerebro-spinal fluid reports, and to Dr. Carey Coombs, under whose care the boy was, for permission to publish and for much help and advice.

*Chart showing the influence of Anti-Meningococcal Serum on the course of the disease.*

### REFERENCES.

[1] Osler, *Boston Med. and Surg. Journ.*, 1898, cxxxix. 641.
[2] Salomon, *Berl. Klin. Woch.*, 1902, xxxix. 1,045.
[3] Andrewes, *Lancet*, 1906, vol. i. 1,172.
[4] Liebermeister, *Münch. Med. Woch.*, 1908, lv., 1,978.
[5] Cecil and Soper, *Arch. Int. Med.*, 1911, viii. 1.
[6] Herrick, *Arch. Int. Med.*, 1919, xxiii. 4.
[7] Findlay, *Journ. R. N. Med. S.*, 1919, v. 198.
[8] Herrick, *Journ. Am. Med. Ass.*, 1918, 70, 227.
[9] Blackfan, *Journ. Am. Med. Ass.*, 1921, lxxvi. 36.
[10] Boulanger-Pilet, *Gaz. des Hôp*, 1923, xcvi. 21 and 53.
[11] Bloedorn, *Am. Journ. Med. Sc.*, 1921, 162, 881.
12 Vignot, *Meningococcémies à forme de fièvre intermittente*, Rennes, 1920.
[13] Liegois et Foulon, *Arch. de Med. et de Pharm Milit.*, 1926, lxxxiv. 1.
[14] Neergard, *Med. Clin. N. Amer.*, 1925–26, ix. 461.
[15] Vaucher et Schmid, *Bull. et Mem. Soc. med. d. hôp. de Par.*, 1922, xlvi.
[16] Netter, *Bull. et Mem. Soc. med. d. hôp. de Par.*, 1923, xlvii.
[17] Gandy et Boulanger-Pilet, *Bull. et Mem. Soc. med. d. hôp. de Par.*, 1922, xlvi.
[18] Debre, Ravine et de Pfeffel, *Soc. de Ped.*, Feb., 1923.
[19] Gandy et Deguinard, *Bull. et Mem. Soc. med. d. hôp. de Par.*, 1922, xlvi.
[20] Lauvergne et Carrot, *Bull. et Mem. Soc. med. d. hôp. de Par.*, 1926, l.
[21] Osler and McCrae, *Modern Medicine*, London, 1925.
[22] Goodall and Washbourn, *Infectious Diseases*, London, 1928, i.
[23] J. D. Rolleston, *Acute Infectious Diseases*, London, 1925.
[24] P. Luton, *These de Paris*, No. 294, 1926.
[25] Hermann and Lifschitz, *Deutsch. Med. Woch.*, 1928, p. 355.
[26] Courtois-Suffit and Garnier, *Bull. et Mem. Soc. med. d. hôp. de Par.*, July, 1926, 2.
[27] Monziols et Loiseleur, *Bull. et Mem. Soc. med. d. hôp. de Par.*, Feb., 1910.
[28] Warfield and Walker, *Bull. of the Ayer Clin. Lab. of the Pennsyl. Hosp. Philad.*, Oct., 1903.
[29] Schottmüller, *Münch. Med. Woch.*, 1905, pp. 1,617, 1,683, 1,729.
[30] Jacobitz, *Zeits. von Hyg.*, April, 1907, 2 and 9.
[31] Portret, "Les Meningococcémies." *These de Paris*, 1912–13, No. 41.

## Figures and Tables

**Figure f1:**